# Supersonic gas flow for preparation of ultrafine silicon powders and mechanochemical synthesis

**DOI:** 10.1098/rsos.181432

**Published:** 2018-11-07

**Authors:** Yang Tao, Jun Lin, Zhao Zhang, Qiuting Guo, Jin Zuo, Changhai Fan, Bo Lu

**Affiliations:** High Speed Aerodynamic Institute, China Aerodynamics Research and Development Center, Mianyang, Sichuan 621000, People's Republic of China

**Keywords:** supersonic gas flow, equipment, ultrafine silicon powders, mechanochemical synthesis

## Abstract

We report the supersonic gas flow for crush and mechanochemical synthesis. The key instrument parameters for production of supersonic particle flow, such as annular nozzle, expansion angle and length of the accelerating duct, are theoretically designed and optimized. Based on the theoretical results, supersonic gas flow equipment is fabricated. The capacity of the present equipment for production of supersonic particle flow is demonstrated by particle image velocimetry measurement, and the maximum transient velocity of the particles achieves as much as 550 m s^−1^. Additionally, the present equipment is applied for continuous and physical preparation of ultrafine Si powders with a high scalability and mechanochemical synthesis of TiO_2_ and TiN_x_ nanopowders at a high production rate.

## 1. Introduction

Mechanochemical synthesis has emerged as an attractive alternative way to synthesize various chemicals and materials such as organic pharmaceuticals cocrystals, nanomaterials, nanocomposites, covalent-organic frameworks, metal-organic frameworks, inorganic catalysts and small organic molecules [1–17]. It features remarkable simplicity, reproducibility, swiftness and cleanness, in which the use of organic solvents is avoided or greatly reduced [18–22]. In mechanochemical procedures, the required energy for cracking of the reactants and activation of chemical reactions is usually offered by mechanical force [23–24]. Generally, the hand grinding and mechanical ball milling are the most common types of energy supply for mechanochemical synthesis [25]. The hand grinding is not tunable and repeatable because different laboratory staff have a distinct hand strength, and the hand grinding speed is variable for every experimental operation. Additionally, the hand grinding is merely suitable for preparation of gram-scale substances. Although the mechanical ball milling is able to overcome these drawbacks, some challenges still remain. For example, the violent ball milling induces an obvious thermal effect, which can break the structure of some substances. Moreover, dropping a small fraction of abraded balls serves as a contaminant for polluting the reaction product. The ball milling has also a low rate of production because it is inherently a batch processing technique. Accordingly, it is the highly urgent need to develop a new mechanochemical method for continuous and scalable mechanochemical synthesis.

The supersonic gas mill, also called the jet mill, can be used for grinding materials, in which solid particles break up upon a collision based on the jet of compressed air or inert gas [26,27]. It has been used across a number of industries, including food and pharmaceutical manufacturing [28–31]. The distinct advantage of the supersonic gas mill product is to avoid the contamination. It is very suitable for continuous and scalable mechanochemical synthesis because the solid particles impact the physical target at a high speed, which is able to effectively convert mechanical energy to trigger a chemical reaction. In addition, the reaction products are easily separated from the gas stream by cyclonic separation in time [29–32]. However, the use of the supersonic gas mill for conducting solvent-free mechanochemical synthesis has not been explored to date. Another important issue is to fabricate the corresponding experimental equipment, which enables the supersonic gas mill-based mechanochemical synthesis for realizing continuous and scalable synthesis.

Herein, we design and fabricate supersonic gas flow equipment. The equipment structure is first investigated theoretically, including annular-flow nozzles, the expansion angle and length of particle accelerating duct. Based on the theoretical results, the supersonic gas flow equipment is fabricated. The speed of the supersonic gas flow equipment is demonstrated by particle image velocimetry. Finally, to prove the practicality of the equipment, it is applied for preparation of ultrafine silicon powders and mechanochemical synthesis of titanium dioxide (TiO_2_) and titanium nitride (TiN_x_) nanopowders for the first time.

## 2. Theoretical and experimental details

### 2.1. Theoretical design of main instrument parameters

#### 2.1.1. Annular nozzle

De Laval nozzle is employed to produce a high speed air flow with a supersonic speed. The isentropic flow was assumed in the annular nozzle. The design Mach number (*M*_design_) depends on the area ratio of exit surface and throat (*A*_ex_/*A*_throat_) according to the following equation:

$$\frac{A_{\mathrm{ex}}}{A_{\mathrm{throat}}}=\frac{1}{M_{\mathrm{design}}}{\left(\frac{2}{\gamma +1}\left(1+\frac{\gamma -1}{2}M_{{\text{design}}^{2}}\right)\right)}^{\gamma +1/2(\gamma -1)},$$

where *γ* is the ratio of specific heats, which is taken to be a constant of 1.4 for the diatomic gas molecules. *A*_ex_ is the area of the exhaust, and *A*_throat_ is the area of the throat.

#### 2.1.2. Particle acceleration duct

The particle acceleration duct is an important component to speed up the particles from a low speed to supersonic speed. In the design process, particles should be guaranteed to have a high speed before reaching the target. Considering the congestion and viscous effect, the length of pipeline should be as short as possible, but the longer acceleration distance is beneficial to the final velocity of the particles. There is a contradiction between the two factors. The parameters of the length and expansion angle of the particle acceleration duct are designed by the gas–solid two-phase flow calculation. Because the volume concentration of the particles in the whole flow is less than 0.1%, the calculation is carried out in the Euler–Lagrangian mode [33]. The detailed numerical method is given in the electronic supplementary material.

### 2.2. Fabrication of supersonic gas flow equipment

We employ the optimized instrument parameters to fabricate the supersonic gas flow equipment. An overall design drawing is achieved, and we further provide it to a local machine factory (Flow Energy Powder Machine Company, Mianyang, China) to fabricate the equipment. The period of production is about 45 days.

### 2.3. Particle image velocimetry measurement

The charge-coupled device camera model 4M3D1 with a resolution of 2048 pixels × 2048 pixels was used (LaVision, Germany), and the maximum sampling rate is 15 frames s^−1^. YAG Double-Cavity Laser was used with the maximum pulse energy of 350 mJ, wavelength of 532 nm, frequency range of 0 ∼ 10 Hz, stability of 3% and pulse width of 5 ns. The system uses an external timing synchronizer for controlling the minimum time to 250 ps. The silicon microparticles (3.9 µm) are employed as the tracking particles.

### 2.4. Preparation of ultrafine silicon powders

For preparation of ultrafine silicon powders, the dry compressed air (*P* = 2.0 MPa, *T* = 298 K) is used to carry Si particles (7.2 µm) to enter the reaction equipment at the rate of 3.6 kg h^−1^. Then, the Si particles are accelerated to high velocities in the range of 450–550 m s^−1^, which sharply collide with the stainless steel target, inducing the size decrease of Si powders. Several products are collected after reaction for 30, 60 and 90 min.

### 2.5. Preparation of TiO_2_ and Ti_x_N nanopowders

Typically, 300 g Ti powder (325 mesh) is added to the reaction equipment using the dry compressed air as the carrier gas. The Ti powder becomes reactive by decreasing its size by the high-speed impacting. After reaction for about 16 min, the self-ignition of the resulting Ti powder occurs. When the reaction fire is extinguished, the product is collected.

### 2.6. Characterization

Powder X-ray diffraction (PXRD) experiments were performed on a Philips X'Pert Pro X-ray diffractometer (PANalytical X'Pert PRO, The Netherlands). Scanning electron microscopy (SEM) images were collected using an S-4300 field emission scanning electron microscope (Hitachi, Japan). The size distribution was carried out with a Malvern laser particle size analyser (Mastersizer 3000, UK).

## 3. Results and discussion

### 3.1. Identification of main instrument parameters

**Figure 1.**
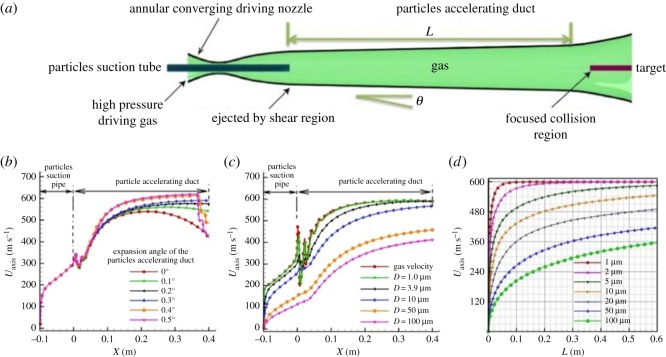
(*a*) Schematic illustration of the key part of reaction equipment. (*b*) Effect of the expansion angle on the central axis velocity (*U*_axis_) of particle accelerating duct. (*c*) Plot of the central axis velocity versus different particle diameters. (*d*) Effect of the length (*L*) of particle accelerating duct on the central axis velocity of different particle sizes.

The annular-flow nozzles and accelerating duct are two critical components of the instrument (figure 1*a*). To accelerate the particle velocity to a higher speed, the particle is concentrated in the target position as much as possible to achieve higher probability of collision. The annular ejector nozzle is designed based on the central crushing material. The nozzle contains both the gradually contracted import and expand export through the air effect. The area ratio of exit surface and throat of the nozzle is about 4.2 to produce a high gas speed with a Mach number of 3. The compressed air, which carries the solid particles, can be accelerated to supersonic speed via the optimization of particle acceleration pipeline length and shape design, and finally collides with the target to realize high-speed collision-promoted mechanical chemical reaction.

The performance of the accelerating duct is dependent on its expansion angle and length. Figure 1*b* shows the expansion angle of the particle accelerating duct on the central axis velocity (*U*_axis_). It can be seen that when the expansion angle is small (below 0.2°), the boundary layer results in the decrease of the actual flow pathway, inhibiting the velocity of air and particles. Nevertheless, if the expansion angle is too large such as 0.5°, the flow will be overexpanded with a normal shock at the end of the duct that may decrease both the speed of gas flow and particles. Based on these considerations, the expansion angle of 0.3° is selected for the particle accelerating duct. Figure 1*c* illustrates the acceleration characteristics of the different diameter (*D*) particles at the expansion angle of 0.3°. All the particles in the range of 1.0–100 µm achieve an acceleration performance, and the small particles reach the velocity as much as 580 m s^−1^. Apart from the expansion angle, the length of the particle accelerating duct is also investigated, as shown in figure 1*d*. When the length is greater than 0.4 m, various solid particles with different sizes in the range of 1.0–100 µm can be accelerated to a high velocity. Accordingly, the length of the particle accelerating duct is maintained at 0.4 m.

### 3.2. Fabrication of the supersonic gas flow equipment and validation of supersonic speed

**Figure 2.**
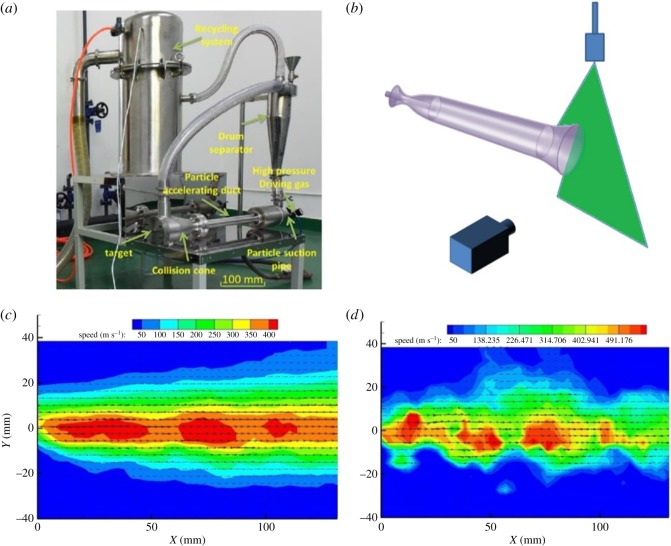
(*a*) Optical image of supersonic gas flow equipment. (*b*) Schematic illustration of the particle image velocimetry (PIV) measurement. (*c*) The average and (*d*) transient velocity distribution diagram of the particles from PIV test.

Based on the optimized instrument parameters, we fabricated the real supersonic gas flow equipment. As shown in figure 2*a*, the equipment includes the following parts: recycling system, drum separator, high-pressure driving gas, particle suction pipe, particle accelerating duct, collision cone and physical target.

To certify the capacity of supersonic gas flow equipment for production of high-speed moving particles, particle image velocimetry (PIV) measurement is conducted, as depicted in figure 2*b*. The time-averaged velocity and transient velocity distributions are shown in figure 2*c*,*d* under the operation pressure of 2 MPa. At the end of the expanded pipe, we can see a series of discs or ring structures. The maximum transient velocity achieves 550 m s^−1^, which is in good agreement with the theoretical results (580 m s^−1^). The average velocity of the gas flow field is about 450 m s^−1^, thanks to the inhomogeneity of the flow field. The high velocity is very beneficial to prepare various products via mechanochemical synthesis.

### 3.3. Ultrafine silicon powders

**Figure 3.**
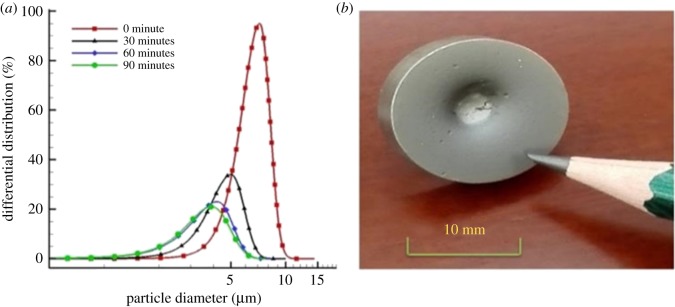
(*a*) Si particle size distribution at different reaction times. (*b*) Optical image of the stainless steel target after reaction.

As a proof of concept, ultrafine Si powders as the model substances are prepared because Si is a key semiconductor in the electron fields. It is necessary to reduce their size before use. We use the fabricated supersonic gas flow equipment to synthesize micro-scale Si particles. As shown in figure 3*a*, the initial Si particles have an average diameter of 7.2 µm, and the diameter gradually decreases with the increase in the reaction time. After reaction for 90 min, the diameter of the Si particles decreases to 3.9 µm, in which 1% of sub-micro Si particles can be obtained. We can calculate the kinetic energy of Si particles according to the PIV results and the theorem of kinetic energy (*E* = (1/2) *mv*^2^), where *E* is the kinetic energy, *m* is the mass of Si particle and *v* is the velocity in the range of 450–550 m s^−1^). The resulting kinetic energy ranges from 101.25 to 151.25 J g^−1^, suggesting the strong interaction between Si particles and physical target. As illustrated in figure 3*b*, the surface of the stainless steel target is full of pits, authenticating that intense impact between Si particles and target occurs. Taken together, these results indicate that this equipment is appropriate for continuous and scalable preparation of ultrafine Si powders.

### 3.4. TiO_2_ and TiN_x_ nanopowders

**Figure 4.**
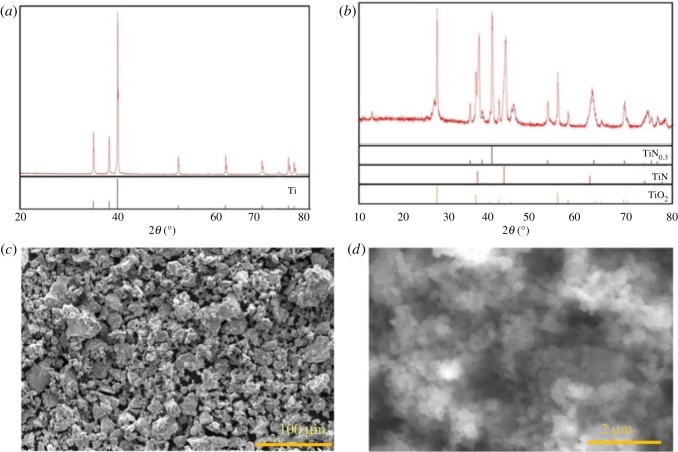
(*a*) XRD pattern of Ti powders and the Ti standard card from JCPDs no. 65-9622. (*b*) XRD pattern of combustion products and the standard cards of TiN_0.3_, TiN and TiO_2_ from JCPDs nos. 41-1352, 65-0414 and 21-1276. SEM image of (*c*) Ti powders and (*d*) the combustion products.

To further testify the validity of this synthesis strategy, the mechanochemical preparation of TiO_2_ and TiN_x_ nanopowders is also performed using Ti powder as the precursor. The self-igniting of small-sized Ti powder is observed. The combustion products are characterized by XRD. As displayed in figure 4*a*, no patterns are ascribed to Ti powders, indicating that Ti is completely consumed during the reaction procedure. The combustion products are identified by the standard XRD card, which consist of TiN, TiN_0.3_ and TiO_2_, as shown in figure 4*b*. On the basis of these results, the reaction equation between Ti powder and air can be given as follows:

$$\mathrm{Ti}+N_{2}+O_{2}\to \mathrm{TiN}+\mathrm{Ti}N_{0.3}+\mathrm{Ti}O_{2}.$$

The morphology of the reaction products is studied by SEM. As shown in figure 4*c*,*d*, compared with Ti raw materials with a microscale diameter, the resulting TiO_2_ and Ti_x_N have an average diameter of 200 nm. These results imply that the TiO_2_ and Ti_x_N nanopowders can be effectively prepared using this equipment.

## 4. Conclusion

We design and fabricate the supersonic gas flow equipment. Some key instruments, including annular nozzle, the expansion angle and length of the accelerating duct, are theoretically designed and optimized. Based on the theoretical results, the supersonic gas flow equipment is fabricated, and the maximum transient velocity of the particles achieves as much as 550 m s^−1^ according to the theoretical values. Importantly, the fabricated equipment has been successfully applied to physically prepare ultrafine silicon powders and mechanochemically synthesize TiO_2_ and TiN_x_ nanopowders at a high production rate. This work opens up a new avenue for mechanochemical synthesis, which has great potential of industrial application in surface modification, nanomaterial preparation and material processing.

## Supplementary Material

Supporting information

## Data accessibility

The authors confirm that all data underlying the findings are fully available without restriction. All relevant data are within the paper and electronic supplementary material.
