# Supplementary material for: Supersonic gas flow for preparation of ultrafine silicon powders and mechanochemical synthesis
Source: R Soc Open Sci. 2018 Nov 7;5(11):181432. doi: 10.1098/rsos.181432 (PMC6281941; doi:10.1098/rsos.181432)
Supplement: Supporting information [file rsos181432supp1.docx]

**Supporting information**

**Supersonic gas flow for preparation of ultrafine silicon powders and mechanochemical synthesis**

Yang Tao^*^, Jun Lin, Zhao Zhang, Qiuting Guo, Jin Zuo, Changhai Fan, Bo Lu

High Speed Aerodynamics Institute, China Aerodynamics Research and Development Center, Mianyang, Sichuan, 621000, China

**Numerical method**

**Gas phase governing equations**

For the three-dimensional unsteady flow, the governing equation is the three-dimensional unsteady Reynolds average Navier-Stokes (NS) equation in the calculated coordinate system, which can be written as^S1^:

|  |  |
| --- | --- |

**Turbulence model**

The turbulence model is used, which is proposed by Menter. In the flow field far from the wall, this model can overcome the shortage that is sensitive to the free stream conditions of traditional model and improves the stability of the model. The model equation with compressible correction of dimensionless form is given directly below^S2^:

| $\begin{matrix} \frac{\partial}{\partial t}\left(\rho k\right)+\frac{\partial}{\partial x_{i}}\left(\rho u_{i}k\right)=\frac{\partial}{\partial x_{i}}\left[\left(\mu+\frac{\mu_{t}}{\sigma_{k}}\right)\frac{\partial k}{\partial x_{i}}\right] \\ +C_{k1}P_{k}-C_{k2}\rho k\omega\end{matrix}$ |
| --- |
| $\begin{matrix} \frac{\partial}{\partial t}\left(\rho\omega\right)+\frac{\partial}{\partial x_{i}}\left(\rho u_{i}\omega\right)=\frac{\partial}{\partial x_{i}}\left[\left(\mu+\frac{\mu_{t}}{\sigma_{\omega}}\right)\frac{\partial\omega}{\partial x_{i}}\right] \\ +C_{\omega1}\frac{\omega}{k}P_{k}-C_{\omega2}\rho\omega^{2} \end{matrix}$ |
| $\mu_{t}=C_{\mu}\frac{\rho k}{\omega}$ |

**Drag law**

Law for drag coefficients (C_D_) is available for the Euler-Lagrange Model. The drag coefficient C_D_ for smooth particles can be taken from the following equation:

$C_{D}=\boldsymbol{a}_{\boldsymbol{1}}\boldsymbol{+}\frac{\boldsymbol{a}_{\boldsymbol{2}}}{\boldsymbol{R}_{\boldsymbol{e}}}\boldsymbol{+}\frac{\boldsymbol{a}_{\boldsymbol{3}}}{{\boldsymbol{R}_{\boldsymbol{e}}}^{\boldsymbol{2}}}$

Where a_1_, a_2_, and a_3_ are the constants that is suitable for several ranges of Re given by Morsi and Alexander ^S3^. For this problem, the particle Mach number is greater than 0.4 at a particle Reynolds number greater than 20. The high-Mach-number correction is used. This drag law is similar to the spherical law ([Equation](file:///I:\Program%20Files\ANSYS%20Inc\v160\commonfiles\help\en-us\help\flu_th\x1-62200016.7.1.html#flu_th_eq9_2_4)up) with corrections^S4^.

**Saffman force**

For this simulation case, the particle’s diameter and velocity gradient are large near the wall boundary in the inject tube, the Saffman lift forces should be considered in the simulation^S5^ Saffman lift force which is from Li and hmadi^S6^ , which is a generalization of the expression provided by Saffman^S7^. The expression is :

$$F=\frac{2Kv^{\frac{1}{2}}\rho d_{ij}}{\rho_{p}d_{p}\left( d_{lk}d_{kl} \right)^{\frac{1}{4}}}(\vec{u}-\vec{u_{p}})$$

where K=2.594 and d_ij_ is the deformation tensor.

**Performing Trajectory Calculations**

The trajectories of solid particles injections are computed when solution iterations were performed. Coupled discrete phase calculations is used for considering their effect on the continuum. This coupled calculation procedure is illustrated in Figure S1. Because the simulation model includes a high mass and/or momentum loading in the discrete phase, the coupled procedure must be followed to include the important impact of the discrete phase on the continuous phase flow field.

**Figure S1**. Coupled discrete phase calculations

S1. Zhu Z Q, Application Computation Fluid Dynamic. The press of Beijing University of Aeronautic and Astronautic, 1998.

S2. Wilcox D C. A Half Century Historical Review of the k- Model. AIAA-91-0615, 1991.

S3. Morsi S. A., Alexander. A. J. "An Investigation of Particle Trajectories in Two-Phase Flow Systems". J. Fluid Mech. 1972;55(2). 193-208.

S4. Clift G, Grace W. "Bubbles, Drops, and Particles". Technical Report.  Academic Press. 1978.

S5. Lun C K, Liu H S. Numerical simulation of dilute turbulent gas-solid flows in horizontal channels. International Journal of Multiphase Flow. 1997;23(4): 605-621.

S6. Li A, Ahmadi G.  Dispersion and Deposition of Spherical Particles from Point Sources in a Turbulent Channel Flow. Aerosol Science and Technology.  1992; 16(2): 209-226

S7. Saffman P G. The Lift on a Small Sphere in a Slow Shear Flow. J. Fluid Mech.  1965: 22(3): 385-400
